# Added Value of Dual-Energy CT in COVID-19 Pneumopathy

**DOI:** 10.5334/jbsr.2598

**Published:** 2021-10-21

**Authors:** Louis Deprez, Yves-Gauthier Boulanger, Julien Guiot

**Affiliations:** 1 Department of Radiology, CHU de Liège, Liège, BE; 2 Avenue de l’Hôpital 1, 4000 Liège, Belgium; 3 Department of Pneumology, CHU de Liège, Liège, BE

**Keywords:** Chest, COVID-19, Pneumonia, Viral, Infectious Diseases, Pulmonary Embolism, Dual-energy Scanner

## Abstract

**Teaching point:** The use of dual-energy instead of conventional single-energy computed tomography pulmonary angiogram can provide additional value concerning the diagnosis of COVID-19 and its complications, especially in the detection of small pulmonary embolism.

## Case

A 74-year-old male was admitted to the emergency room for acute dyspnea and acute chest pain. Medical history involved a fall two days before admission, with progressively increasing right chest pain and apparition of productive cough with white sputum. Clinical parameters showed hypoxemia (ambient air oxygen saturation 82%), tachycardia (95 beats per minute [BPM]) and fever (38°C). Biology showed neutrophilic leukocytosis (22 000 white blood cells/mm^3^) and an elevated C-reactive protein blood level (208,8 mg/L).

The initial non-contrast-enhanced chest computed tomography (CT) demonstrated multiple right rib fractures, right hemothorax, and lung condensation with bronchial wall thickening in the left lower lobe, which was diagnosed as community acquired pneumonia (not shown).

Nasal swab Reverse Transcriptase Polymerase Chain Reaction (RT-PCR testing) was negative for SARS-CoV-2. The follow-up chest CT at one week showed a regression of the left lower lobe pneumonia.

**Figure 1:**
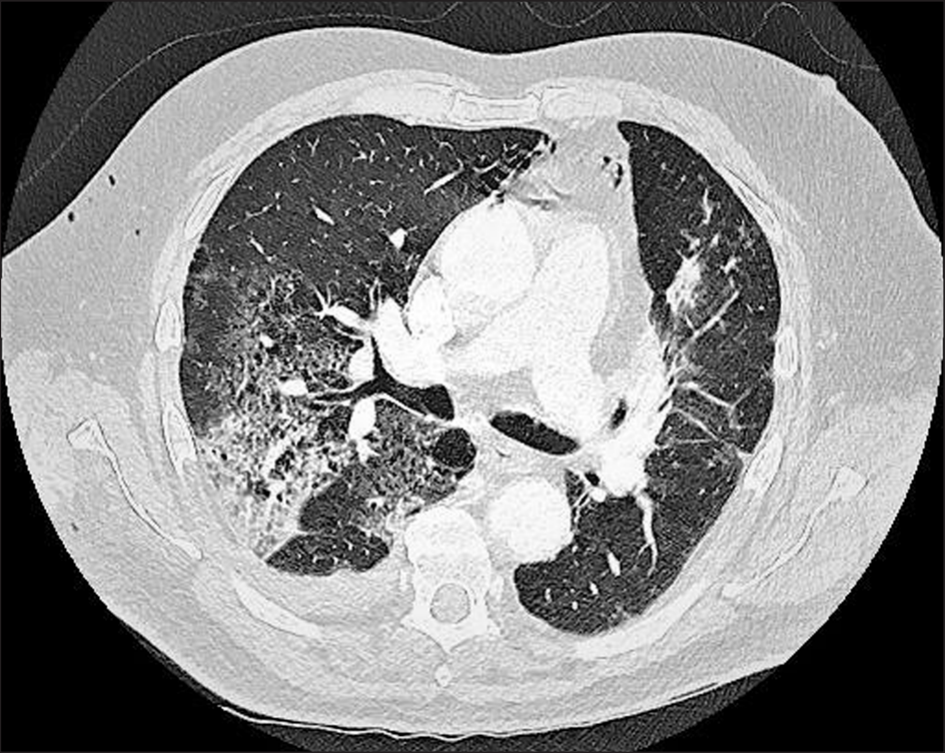


Two days later, the patient suffered from majorated hypoxemia (88% oxygen saturation with 4 L/min of O_2_) and tachycardia (105 BPM), and a CT pulmonary angiogram (CTPA) was performed using a dual-energy scanner. It showed primarily ground-glass opacities with intralesional reticulations and consolidation in the right upper lobe, right lower lobe and lingula, suggestive of COVID-19 pneumonia (***Figure 1***).

**Figure 2:**
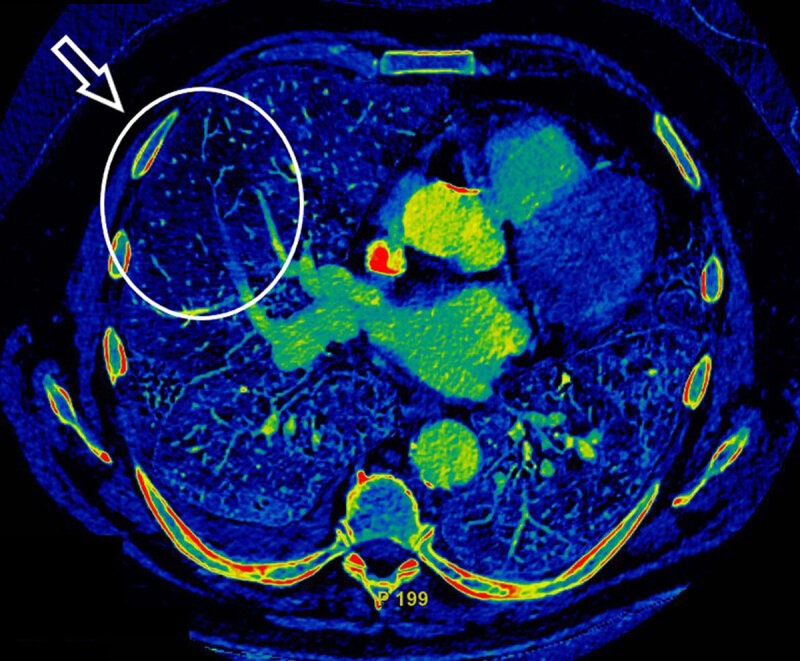


**Figure 3:**
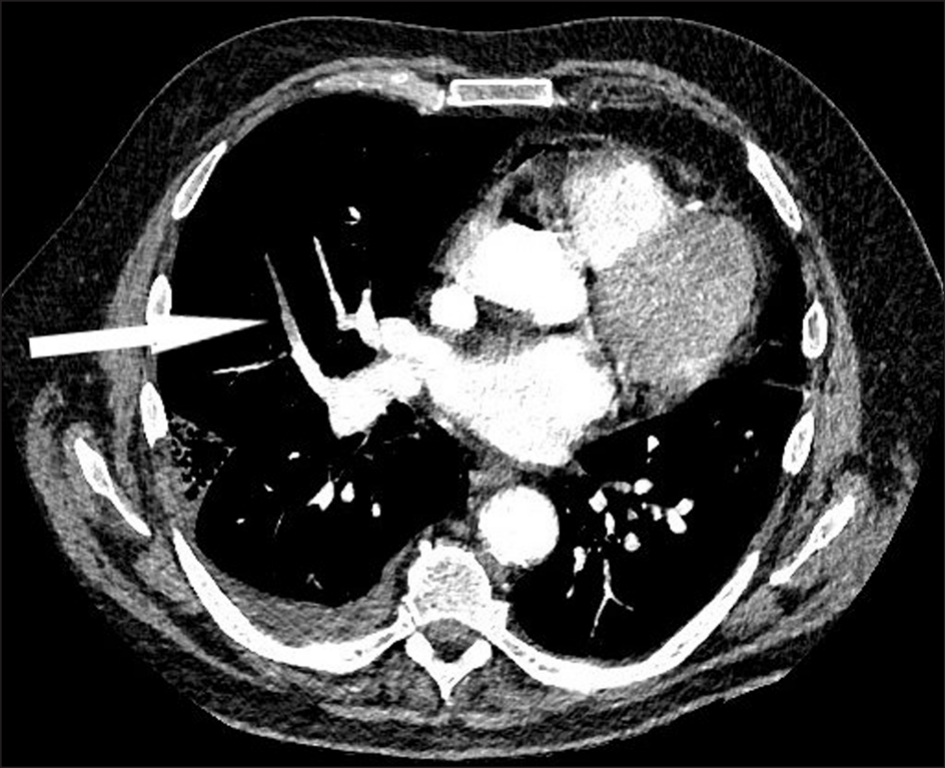


While those lesions did not completely explain the patient’s major hypoxemia, the iodine cartography (***Figure 2***, white circle) demonstrated a perfusion defect in the right middle lobe, with a corresponding distal pulmonary embolism on the CT image (***Figure 3***, white arrow), associated with indirect signs of pulmonary hypertension (right ventricle/left ventricle ratio >1 and increased pulmonary arteries diameter) (not shown).

Another RT-PCR was then performed and formally confirmed the diagnosis of COVID-19 infection.

## Comment

While the lung parenchymal part of COVID-19 infection is largely recognized, its involvement in endothelial injury also seems to be a major factor of morbi-mortality [1]. Indeed, the vascular component of the disease leads to a high risk of pulmonary embolism, invisible on plain CT and underdiagnosed with conventional CTPA, mainly regarding distal emboli which seems to be more common than their proximal counterparts. Taking all those parameters into account, we think that the use of dual-energy CTPA scan (especially with the use of a perfusion cartography) could help diagnose small embolic complications, which are relevant to the clinical course of the pathology and its treatment.

## References

[1] Roncon L, Zuin M, Barco S, et al. Incidence of acute pulmonary embolism in COVID-19 patients: Systematic review and meta-analysis. Eur J Intern Med. 2020; 82: 29–37. DOI: 10.1016/j.ejim.2020.09.006 32958372 PMC7498252

